# CT-based radiomics for predicting lymph node metastasis in esophageal cancer: a systematic review and meta-analysis

**DOI:** 10.3389/fonc.2024.1267596

**Published:** 2024-03-19

**Authors:** Liangsen Liu, Hai Liao, Yang Zhao, Jiayu Yin, Chen Wang, Lixia Duan, Peihan Xie, Wupeng Wei, Meihai Xu, Danke Su

**Affiliations:** ^1^ Department of Medical Imaging Center, Guangxi Medical University Cancer Hospital, Nanning, China; ^2^ Department of Nuclear Medicine, The Fifth Affiliated Hospital of Guangxi Medical University, Nanning, China; ^3^ Department of Radiology, The Fifth Affiliated Hospital of Guangxi Medical University, Nanning, China; ^4^ Department of Radiology, The Second Affiliated Hospital of Guangxi Medical University, Nanning, China

**Keywords:** esophageal cancer, lymph node metastasis, computerized tomography, radiomics, diagnosis, meta-analysis

## Abstract

**Objective:**

We aimed to evaluate the diagnostic effectiveness of computed tomography (CT)-based radiomics for predicting lymph node metastasis (LNM) in patients diagnosed with esophageal cancer (EC).

**Methods:**

The present study conducted a comprehensive search by accessing the following databases: PubMed, Embase, Cochrane Library, and Web of Science, with the aim of identifying relevant studies published until July 10th, 2023. The diagnostic accuracy was summarized using the pooled sensitivity, specificity, positive likelihood ratio (PLR), negative likelihood ratio (NLR), diagnostic odds ratio (DOR), and area under the curve (AUC). The researchers utilized Spearman’s correlation coefficient for assessing the threshold effect, besides performing meta-regression and subgroup analysis for the exploration of possible heterogeneity sources. The quality assessment was conducted using the Quality Assessment of Diagnostic Accuracy Studies-2 and the Radiomics Quality Score (RQS).

**Results:**

The meta-analysis included six studies conducted from 2018 to 2022, with 483 patients enrolled and LNM rates ranging from 27.2% to 59.4%. The pooled sensitivity, specificity, PLR, NLR, DOR, and AUC, along with their corresponding 95% CI, were 0.73 (0.67, 0.79), 0.76 (0.69, 0.83), 3.1 (2.3, 4.2), 0.35 (0.28, 0.44), 9 (6, 14), and 0.78 (0.74, 0.81), respectively. The results demonstrated the absence of significant heterogeneity in sensitivity, while significant heterogeneity was observed in specificity; no threshold effect was detected. The observed heterogeneity in the specificity was attributed to the sample size and CT-scan phases (P < 0.05). The included studies exhibited suboptimal quality, with RQS ranging from 14 to 16 out of 36. However, most of the enrolled studies exhibited a low-risk bias and minimal concerns relating to applicability.

**Conclusion:**

The present meta-analysis indicated that CT-based radiomics demonstrated a favorable diagnostic performance in predicting LNM in EC. Nevertheless, additional high-quality, large-scale, and multicenter trials are warranted to corroborate these findings.

**Systematic Review Registration:**

Open Science Framework platform at https://osf.io/5zcnd.

## Introduction

Esophageal cancer (EC), a prevalent and deadly neoplasm, has been identified as the seventh most commonly diagnosed cancer globally in 2020, with 604,000 new cases reported and the sixth leading cause of death, with 544,000 fatalities (1). Patients with EC are often diagnosed at advanced stages, making them unsuitable for surgery and leading to a poor prognosis with low 5-year survival rates of only 20%-30% (2–4). Lymph node metastasis (LNM) has been identified as a vital prognostic determinant for patient survival (5–7). The eighth edition of the AJCC’s International Staging Standard for Esophageal Cancer introduced a clinical staging system based on preoperative imaging; it incorporated the count of lymph node metastases in postoperative staging (8, 9). While pathological findings continue to be the gold standard for diagnosing LNM, lymph node biopsy represents an invasive procedure with a non-trivial incidence of complications (10). Therefore, accurate evaluation of lymph node status using non-invasive imaging methods is imperative for making informed treatment decisions and essential for precise prognostication.

The computed tomography (CT) scans is a widely used non-invasive imaging technique for acquiring preoperative and postoperative tumor-related data to assess lymph node status in EC patients (11). However, relying solely on morphological standards, including short diameter and shape measured by physicians with varying levels of clinical diagnostic expertise, conventional CT scans are inadequate for accurately identifying LNM. The accuracy of conventional CT scans for precise detection is suboptimal, with sensitivity ranging from 37.3% to 67.2% and specificity ranging from 63.9% to 96.4% (12). Furthermore, these criteria have limitations culminating in a markedly low diagnostic accuracy for normal-sized lymph nodes. Consequently, conventional evaluation of lymph node status through CT scans remains challenging.

Radiomics is an innovative technique that swiftly extracts numerous quantitative features from conventional medical images using high-throughput computation, yielding invaluable information for diagnostic and prognostic purposes (13–15). In recent years, radiomics has been extensively employed in detecting, grading, assessing the therapeutic response, and prognostic evaluation for patients with EC (16–20). CT-based radiomics have been utilized to predict LNM in patients with EC (11, 21–23). These findings suggest that this approach has great potential as an accurate and reproducible tool for non-invasive preoperative evaluation of LNM. Radiomics techniques offer a promising solution that overcomes some of the limitations of conventional CT imaging. However, owing to variations in imaging protocols, study design, sample size, modeling techniques, and software used for radiomics analysis across various studies on EC, the reported diagnostic efficacy of radiomics in preoperative identification of LNM has demonstrated significant variability. These inconsistencies have led to uncertainty regarding the effectiveness of using radiomics for this purpose in clinical practice. Therefore, we aimed to conduct a comprehensive meta-analysis that thoroughly assesses the diagnostic accuracy of CT-based radiomics in predicting LNM in individuals diagnosed with EC.

## Materials and methods

This study followed the guidelines of Preferred Reporting Items for Systematic Reviews and Meta-Analyses (PRISMA) (24). The protocol for this review has been registered on the Open Science Framework (OSF) platform, with the registration link available at https://osf.io/5zcnd.

### Literature search

In attempting to comprehensively identify all studies that may be related to our question, an independent search was conducted by two authors (L.S.L. and Y.Z.) in four databases, namely PubMed, Embase, Web of Science, and Cochrane Library, which was limited to studies published until July 10th, 2023. Various keywords, including “artificial intelligence,” “machine learning,” “radiomics,” “deep learning,” “esophageal neoplasms,” “esophageal cancer,” “lymph node metastasis,” “lymph node,” and “LNM” were used for the search. MeSH terms and variations of each keyword were utilized during the search to ensure inclusivity. Any disagreements during the literature selection process were resolved through discussion and consensus among the research team, with the assistance of a designated third-party reviewer (H.L.).

### Study selection

The selected studies had to meet specific criteria: (1) original research studies with sample sizes greater than 40; (2) histopathological diagnosis of EC and LNM; (3) LNM detected using CT-based radiomics, and (4) data sufficient for reconstructing 2×2 contingency table, aiming at determining diagnostic sensitivity and specificity. Meanwhile, the criteria used to exclude ineligible studies were: (1) reviews, case reports, consensus statements, guidelines, animal studies, letters, and editorials, and (2) multiple studies using the same study population (in such cases, the most recent or comprehensive report was also included).

### Quality assessment

The quality assessment and data extraction were independently evaluated by two reviewers, L.S.L. and Y.Z. Any disagreements were resolved by the third reviewer, H.L. Four domains of the Quality Assessment of Diagnostic Accuracy Studies-2 (QUADAS-2) were customized to evaluate the potential bias in the selected studies, including patient selection, index testing, reference standards, and flow and timing (25). The researchers utilized the Radiomics Quality Score (RQS) to evaluate the methodological quality of the included studies. The RQS comprises five components: imaging protocol, feature extraction from radiological images, data modeling, model validation, and data sharing (13). Additional details can be found in the **Supplementary Table S1**. The concordance between the two primary reviewers was determined through the calculation of the intra-class correlation coefficient (ICC). The ICC values were classified as excellent (≥ 0.85), good (0.70–0.84), moderate (0.55–0.69), and weak or poor (≤ 0.54) (26).

### Data extraction

All pertinent data was acquired from the entirety of the incorporated full-text articles. The information that was obtained through extraction were: first author, publication year, country, study type, total number of patients and LNM cases, CT machine type, segmentation details, feature selection method, algorithms, information about radiomics and deep learning, data source (single or multiple institutions), sensitivity, and specificity. The researchers calculated the numbers of true positive/negative and false positive/negative cases using the reported sensitivity, specificity, LNM-present, and LNM-absent values in each study. If a single study presented multiple models derived from the same patient cohort, only the model demonstrating superior diagnostic accuracy in the validation cohort (or the training cohort if a validation cohort was unavailable) was considered for inclusion in this meta-analysis (27).

### Statistical analysis

Stata 16.0, Meta-Disc 1.4, and Review Manager 5.3 were employed for statistical analysis of meta-analysis. Diagnostic accuracy was evaluated by pooling sensitivity, specificity, positive likelihood ratio (PLR), negative likelihood ratio (NLR), and diagnostic odds ratio (DOR) with their respective 95% CI. The summary receiver operating characteristic curve (SROC) and its corresponding area under the curve (AUC) were used to summarize the findings. Heterogeneity assessment of the studies incorporated in the analysis was performed through Cochran’s Q-test and Higgins’ I2 test. A P < 0.05 (Cochran’s Q-test) or a Higgins’ I2 value >50% indicated significant heterogeneity between the studies (28). Sensitivity analysis were conducted by systematically eliminating individual studies from the meta-analysis calculations to evaluate their influence on the overall estimation. Deeks’ test assessed the publication bias by analyzing the effective sample size funnel plot (29).

### Clinical utility

The study employed Fagan plot analysis for assessing the clinical effectiveness of CT-based radiomics in predicting LNM. This method calculated the LNM post-test probability (P-post) based on the pre-test probabilities (P-pre), signifying a suspicion of LNM (30).

## Results

### Study selection

**Figure 1:**
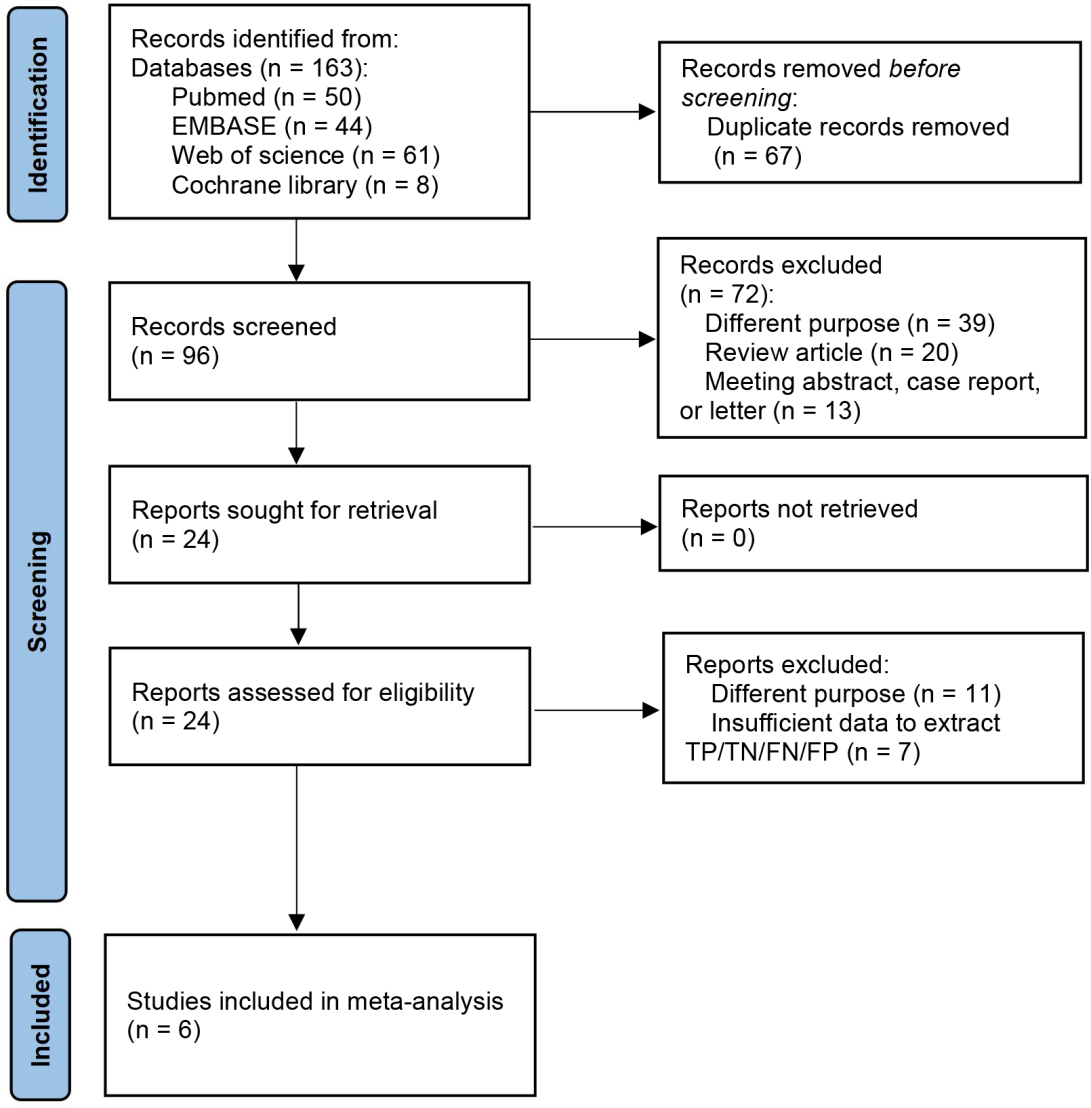
Flow diagram of study selection for meta-analysis according to PRISMA.

By using the aforementioned search strategy, 163 studies were initially identified; only 96 remained after duplicate removal. After reviewing the titles and abstracts, only 24 studies were deemed eligible for further analysis and potential inclusion. After carefully reading the full-text articles, six studies (21, 31–35) were considered eligible for inclusion. In contrast, seven were excluded due to insufficient data, and eleven did not meet the intended purpose of investigating radiomics for predicting LNM (**Figure 1**).

### Features of the enrolled studies

**Table 1** lists an overview of the six enrolled studies spanning from 2018 to 2022. A total of 483 patients were enrolled, with LNM rates varying between 27.2% to 59.4%. The included studies were conducted retrospectively and exclusively in China. Additionally, all the studies were based on single-center data. Three studies focused on esophageal squamous cell carcinoma, while the remaining three did not specify the cancer subtype. Manual segmentation was utilized for radiomics analysis in all the studies. Furthermore, only one study combined feature extraction with deep learning methods, while the other five exclusively relied on radiomics. The feature selection methods employed in radiomics analysis included the least absolute shrinkage and selection operator (LASSO), t-test, analysis of variance, and ridge regression. In terms of the radiomics diagnostic model developed using machine learning algorithms, logistic regression (LR) was used in four studies, while random forest (RF) and support vector machine (SVM) were used in one study each.

**Table 1:** Key characteristics of included studies in the meta-analysis.

Author	Year	Country	Study design	Tumor type	Sample size	LNM,n(%)	Scanner	Phases	Segmentation method	ROI	Feature selection method	Algorithms	Combine deep learning (Yes/No)	Data source
Chen	2022	China	Re	ESCC	92	46(50.0)	NA	CE	manual	2D	LASSO, t-test	RF	YES	Single institution
Li	2021	China	Re	EC	60	25(41.7)	Siemens	NCE, CE	manual	3D	LASSO, Analysis of variance	LR	NO	Single institution
Ou	2021	China	Re	ESCC	101	60(59.4)	GE	CE	manual	3D	LASSO, t-test, Mann-Whitney U test	LR	NO	Single institution
Peng	2022	China	Re	ESCC	81	41(50.6)	GE	NCE, CE	manual	3D	LASSO	LR	NO	Single institution
Shen	2018	China	Re	EC	57	19(33.3)	GE, Phillips	CE	manual	3D	LASSO、Ridge regression	LR	NO	Single institution
Yu	2021	China	Re	EC	92	25(27.2)	GE	NCE	manual	3D	LASSO	SVM	NO	Single institution

NA, not available; Re, retrospective; ESCC, esophageal squamous cell carcinoma; EC, esophageal carcinoma; LNM, lymph node metastasis; TP, true positive; FP, false positive; FN, false negative; TN, true negative; CE, contrast-enhanced; NCE, non-contrast-enhanced; ROI, region of interest; 2D, two−dimensional; 3D, three−dimensional; LASSO, least absolute shrinkage and selection operator; LR, logistic regression; RF, random forest; SVM, support vector machine.

### Quality assessment and publication bias

**Figure 2:**
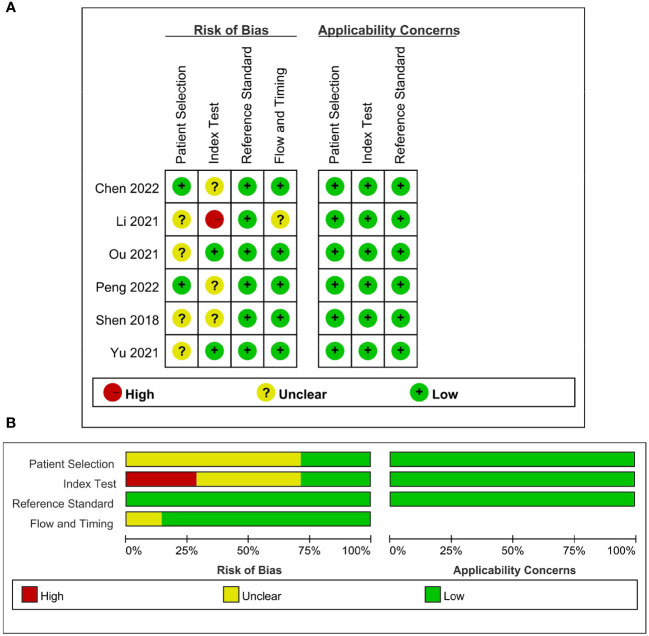
Quality assessment of included studies according to Quality Assessment of Diagnostic Accuracy Studies-2 (QUADAS-2) criteria. **(A)** Individual studies, **(B)** summary.

**Figure 3:**
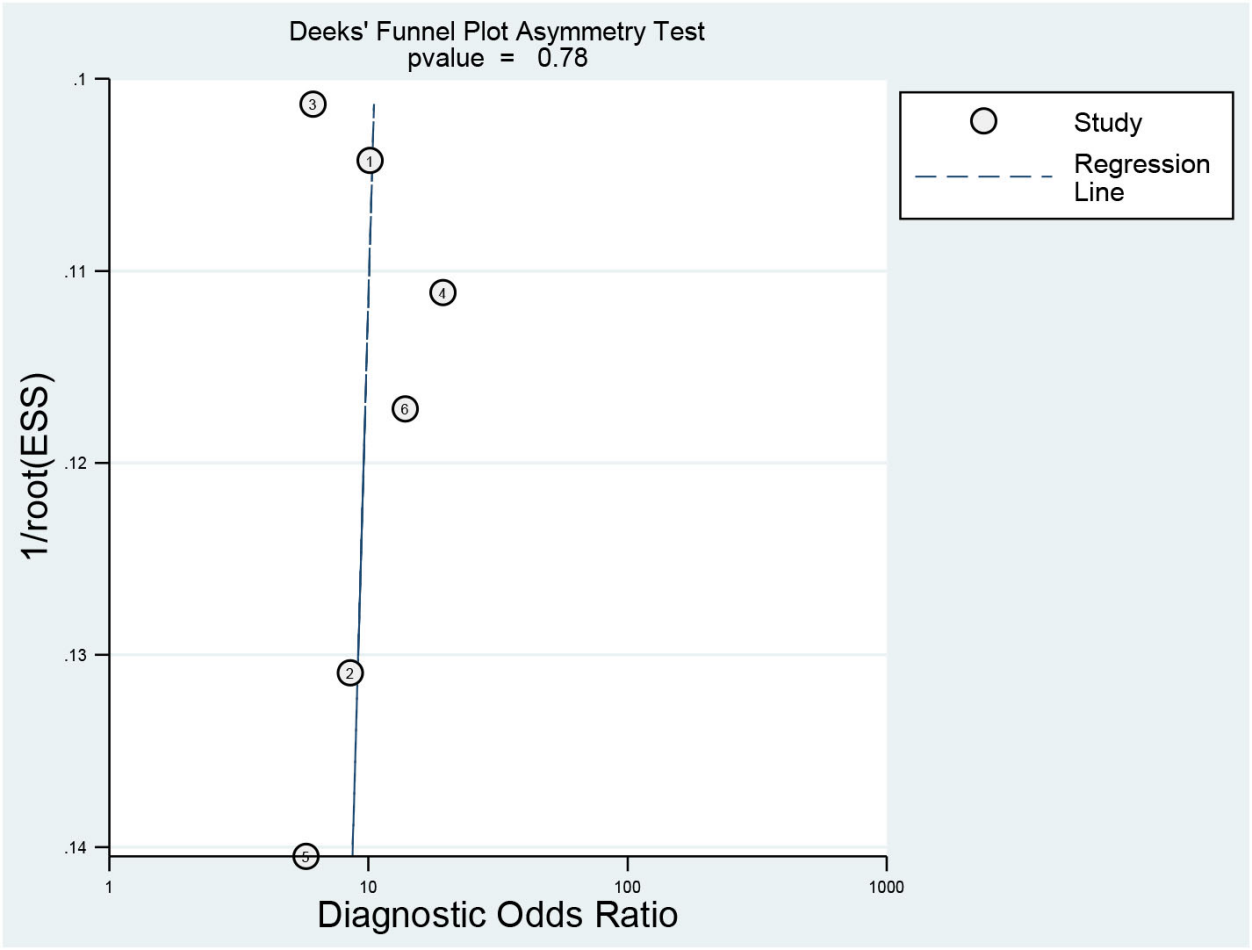
Deeks’ funnel plot asymmetry test for publication bias.

The detailed assessments of RQS and QUADAS-2 for each study are provided in **Supplementary Tables S2** and **S3**. The agreement between primary reviewers was excellent, with ICC values of 0.94 (95% CI 0.64-0.99) for RQS and 0.92 (95% CI 0.56-0.99) for QUADAS-2. The RQS varied between 14 and 16 across studies, with two studies (33, 34) achieving the highest RQS percentage at 44.4%. However, all the selected studies lacked the use of phantoms to evaluate robustness, prospective research design, discussion of potential biological correlates, or comprehensive cost-effectiveness analysis. The qualitative assessment using the QUADAS-2 tool indicated that most studies had a low risk of bias and minimal concerns regarding their applicability (**Figure 2**). Deeks’ funnel plot analysis revealed no evidence of publication bias, suggesting a low risk of bias among the included studies. (**Figure 3**; P = 0.78).

### Diagnostic accuracy of CT-based radiomics

**Figure 4:**
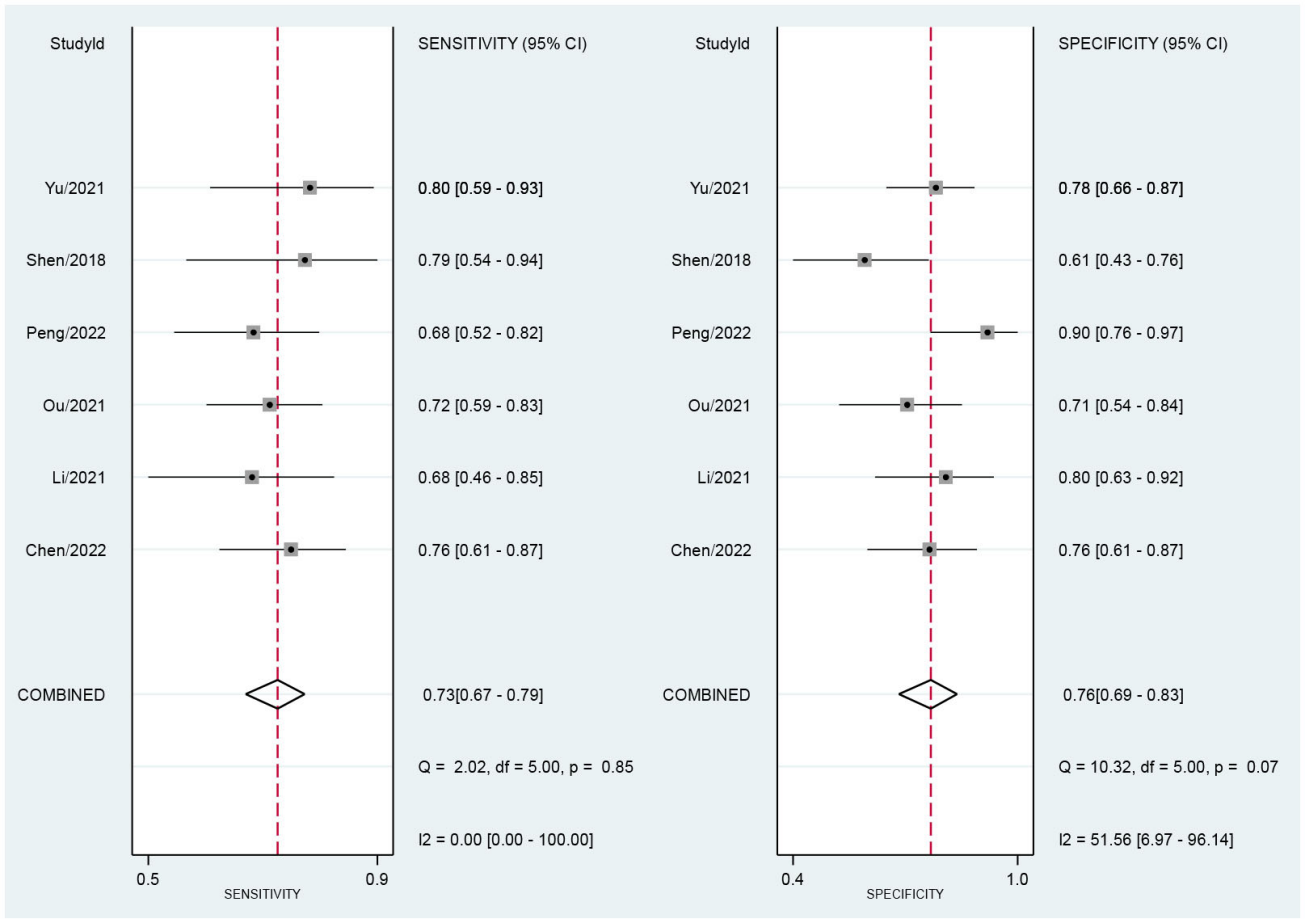
Forest plot of sensitivity and specificity based on radiomics for predicting LNM in esophageal carcinoma.

**Figure 5:**
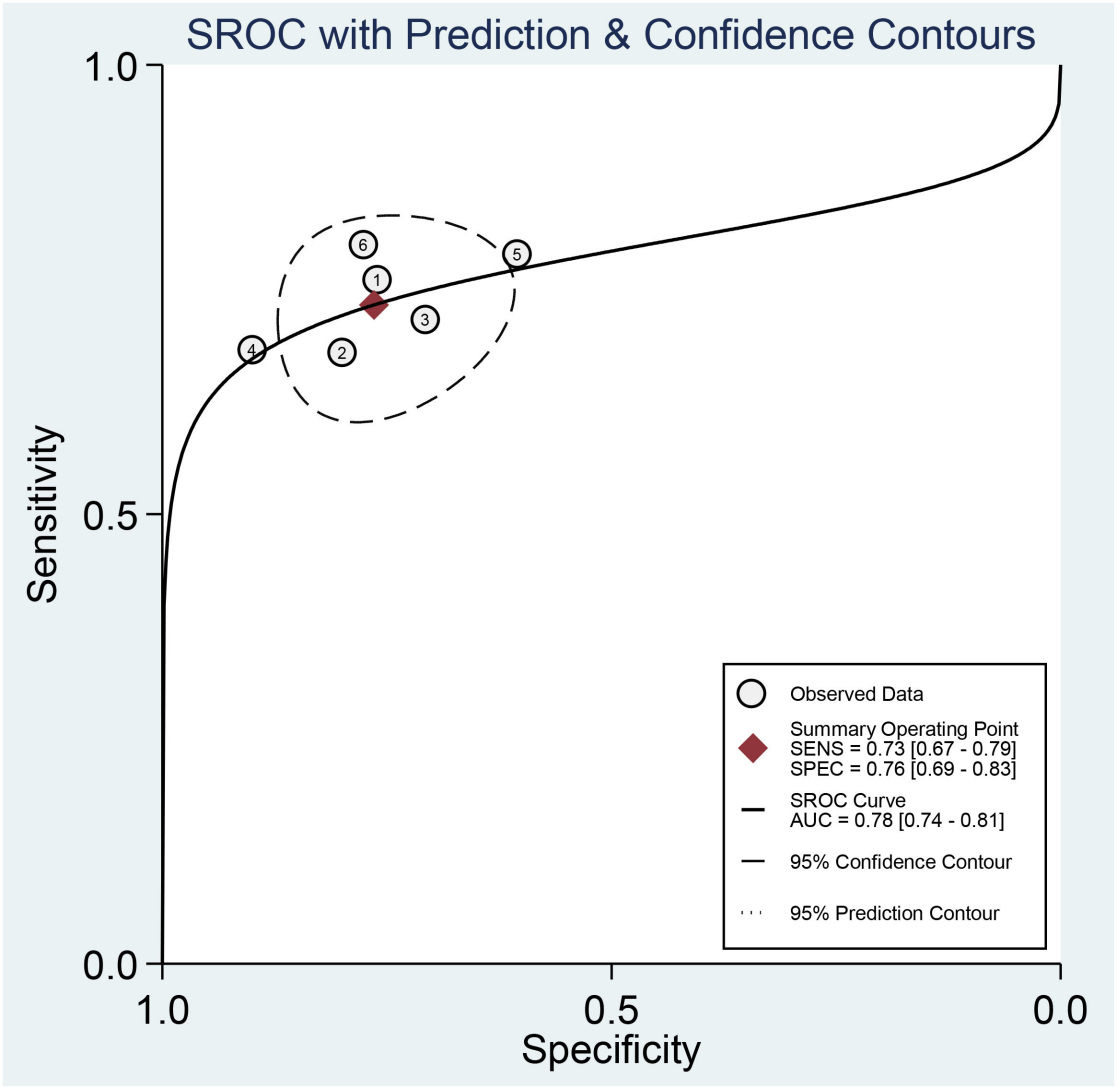
Summary receiver operating characteristic curves (SROC) based on radiomics for predicting LNM in esophageal carcinoma.

Across all six selected studies, the pooled sensitivity and specificity (as displayed by the forest plots in **Figure 4**) for the CT-based radiomics in evaluating LNM in EC were determined to be 0.73 (95% CI, 0.67-0.79) and 0.76 (95% CI, 0.69-0.83), respectively. The PLR, NLR, and DOR were found to be 3.1 (95% CI, 2.3-4.2),  0.35 (95% CI, 0.28-0.44), and 9 (95% CI, 6-14), respectively. Furthermore, the SROC analysis yielded an AUC of 0.78 (95% CI, 0.74-0.81), indicating significant overall diagnostic efficacy (**Figure 5**).

### Heterogeneity assessment

**Figure 4** presents the forest plot, which indicates no significant heterogeneity among the studies included in the research when considering sensitivity (P=0.85; I2 = 0). However, specificity exhibited a significant heterogeneity (P=0.07) with a moderate level of heterogeneity indicated by an I2 value of 51.56% (95% CI, 6.97%-96.14%). The Spearman’s correlation coefficient was 0.543, with a non-significant P-value of 0.266, suggesting the absence of a threshold effect.

### Meta-regression

The sources of heterogeneity were identified through the application of univariate meta-regression analysis. **Table 2** presents the results of both subgroup analysis and univariate meta-regression, revealing that several categories, including tumor type, LNM ratio, feature selection method, and algorithms, significantly contributed to the heterogeneity observed in the pooled sensitivity (P < 0.05). Moreover, the sample size and CT-scan phases used were identified as contributors to the heterogeneity in specificity analysis (P < 0.05). The forest plot is presented in **Supplementary Figure S1**.

**Table 2:** Results of univariate meta-regression and subgroup analyses.

Subgroup	Category	Studies (n)	Sensitivity (95%CI)	P value	Specificity (95%CI)	P value
Tumor type	ESCC	3	0.72 (0.65-0.79)	0.02	0.79 (0.70-0.88)	0.16
	EC	3	0.75 (0.65-0.86)		0.74 (0.64-0.83)	
Sample size	≥90	3	0.75 (0.67-0.82)	0.09	0.75 (0.66-0.85)	0.04
	<90	3	0.71 (0.62-0.81)		0.78 (0.68-0.88)	
LNM ratio	≥50%	3	0.72 (0.65-0.79)	0.02	0.79 (0.70-0.88)	0.16
	<50%	3	0.75 (0.65-0.86)		0.74 (0.64-0.83)	
Scanner	GE	3	0.72 (0.64-0.80)	0.13	0.80 (0.71-0.88)	0.53
	Other	2	0.73 (0.60-0.86)		0.70 (0.57-0.84)	
Phases	CE	3	0.74 (0.67-0.82)	0.06	0.70 (0.62-0.78)	0.00
	Other	3	0.71 (0.62-0.81)		0.82 (0.75-0.88)	
ROI	2D	1	0.76 (0.63-0.89)	0.20	0.76 (0.60-0.93)	0.27
	3D	5	0.72 (0.66-0.79)		0.76 (0.69-0.84)	
Feature selection method	LASSO	2	0.73 (0.62-0.84)	0.05	0.83 (0.74-0.91)	0.27
	Other	4	0.73 (0.66-0.81)		0.72 (0.64-0.80)	
Algorithms	LR	4	0.71 (0.64-0.79)	0.01	0.76 (0.67-0.85)	0.06
	Other	2	0.77 (0.68-0.87)		0.77 (0.66-0.88)	
Combine deep learning	Yes	1	0.76 (0.63-0.89)	0.20	0.76 (0.60-0.93)	0.27
	No	5	0.72 (0.66-0.79)		0.76 (0.69-0.84)	

ESCC, esophageal squamous cell carcinoma; EC, esophageal carcinoma; LNM, lymph node metastasis; CE, contrast-enhanced; NCE, non-contrast-enhanced; ROI, region of interest; 2D, two−dimensional; 3D, three−dimensional; LASSO, least absolute shrinkage and selection operator; LR, logistic regression.

### Subgroup analysis

Studies on esophageal squamous cell carcinoma (n=3) demonstrated equivalent sensitivity (72%, 95% CI, 65-79 vs. 75%; 95% CI, 65-86) and higher specificity (79%, 95% CI, 70-88 vs. 74%; 95% CI, 64-83) compared to studies on esophageal carcinoma (n=3). Studies with ≥90 patients (n=3) showed equivalent sensitivity (75%, 95% CI 67-82 vs. 71%, 95% CI 62-81) and specificity (75%, 95% CI 66-85 vs. 78%, 95% CI 68-88) compared to studies with <90 patients (n=3). Studies with an LNM ratio ≥50% (n=3) had equivalent sensitivity (72%, 95% CI, 65-79 vs. 75%; 95% CI, 65-86) and higher specificity (79%, 95% CI, 70-88 vs. 74%; 95% CI, 64-83) when compared to studies with an LNM ratio of <50% (n=3).

Three studies using only General Electric(GE) equipment showed similar sensitivity (72%, 95% CI, 64-80 vs. 73%, 95% CI, 60-86) and higher specificity (80%, 95% CI, 71-88 vs. 70%, 95% CI, 57-84) to two studies using other equipment. Three studies using contrast-enhanced CT only had equivalent sensitivity (74%, 95% CI, 67-82 vs. 71%; 95% CI, 62-81) and lower specificity (70%, 95% CI, 62-78 vs. 82%; 95% CI, 75-88) than three studies using other methods. In terms of ROI selection, only one study using the 2D method showed similar sensitivity (76%, 95% CI, 63-89 vs. 72%, 95% CI, 66-79) and specificity (76%, 95% CI, 60-93 vs. 76%, 95% CI, 69-84) compared to five studies that employed the 3D method. Two studies using the LASSO method had similar sensitivity (73%, 95% CI, 62-84 vs. 73%, 95% CI, 66-81) and higher specificity (83%, 95% CI, 74-91 vs. 72%, 95% CI, 64-80) compared to four other studies using different methods. Studies (n=4) utilizing LR for their model had lower sensitivity (71%, 95% CI, 64-79 vs. 77%, 95% CI, 68-87) and equivalent specificity (76%, 95% CI, 67-85 vs. 77%, 95% CI, 66-88) compared to studies (n=2) using different algorithms. A study that combined deep learning features (n=1) showed similar sensitivity (76%, 95% CI, 63-89 vs. 72%, 95% CI, 66-79) and specificity (76%, 95% CI, 60-93 vs. 76%, 95% CI, 69-84), when compared to studies solely utilizing radiomics (n=5).

### Sensitivity analysis

**Supplementary Table S1** lists the sensitivity analysis results for each of the six chosen studies. Our findings demonstrated the robustness of results as no significant changes were observed when excluding each study individually; this suggested that any particular study did not significantly influence the overall outcome.

### Clinical utility

**Figure 6:**
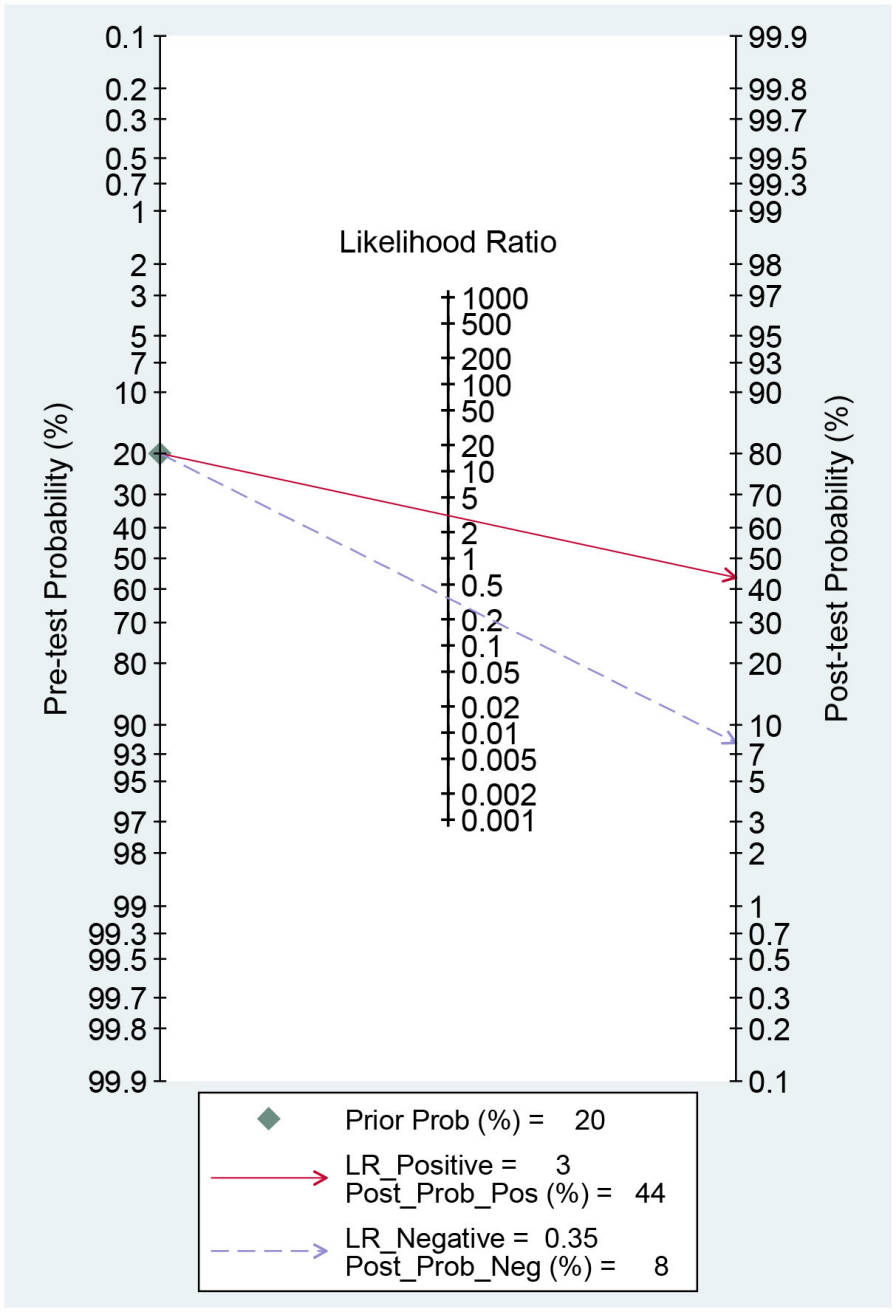
Fagan plots for assessing clinical utility.

A CT-based radiomics model can substantially increase the P-post from 20% to 44% with a PLR of 3 for positive pre-tests. Conversely, it can decrease the P-post to 8% with an NLR of 0.35 for negative pre-tests (**Figure 6**). This section may be divided by subheadings. It should provide a concise and precise description of the experimental results, their interpretation, as well as the experimental conclusions that can be drawn.

## Discussion

According to our current awareness, this study represented the first systematic review and meta-analysis investigating the efficacy of CT-based radiomics in assessing LNM among patients diagnosed with EC. Our findings demonstrated that the pooled sensitivity, specificity, and AUC for CT-based radiomics were 0.73 (95% CI, 0.67-0.79), 0.76 (95% CI, 0.69-0.83), and 0.78 (95% CI, 0.74-0.81), respectively, showcasing its significantly effective performance. The good sensitivity was crucial for accurately identifying most patients with LNM, thus reducing the chances of missed diagnoses. Furthermore, the high specificity was key in lowering false-positive rates, offering a reliable way to rule out LNM in patients and helping clinicians avoid unnecessary treatments and their potential complications. The inclusion of positive and negative likelihood ratios (PLR and NLR) of 3.1 and 0.35, respectively, further improved the diagnostic utility of CT-based radiomics. A PLR of 3.1 meant that patients with LNM were over three times more likely to have a positive test result than those without, greatly increasing the accuracy of identifying affected individuals. On the other hand, an NLR of 0.35 indicated that a negative result significantly reduced the chance of LNM by 65%, lowering the risk of false negatives. These ratios adjusted the post-test probability based on the initial probability, with a positive test increasing the probability from 20% to 44%, and a negative test decreasing it to about 8%. Such adjustments significantly changed how test results were interpreted clinically, boosting the predictive accuracy. Therefore, our study validated the precision and effectiveness of CT-based radiomics in predicting LNM in EC, facilitating personalized treatment plans. By precisely detecting LNM and improving cancer staging, it facilitated more personalized care, optimizing treatment for high-risk patients while avoiding unnecessary interventions for others.

A moderate degree of heterogeneity in terms of specificity was indicated by the meta-analysis of the studies included, which is worth noting. To determine the possible sources of heterogeneity, a univariate meta-regression analysis and subgroup analysis were conducted, as the Spearman’s correlation coefficient test revealed that heterogeneity was not associated with threshold effects. The results suggested that the heterogeneity in specificity could be attributed to the utilization of different CT scan phases and variations in sample size. However, upon conducting a subgroup analysis, it was observed that the sensitivity and specificity were not significantly affected by these factors. It is important to acknowledge that due to the variations in methodologies employed among the included studies, it was challenging to identify all the factors contributing to the observed heterogeneity. Interestingly, while there was no heterogeneity in the pooled sensitivity across all studies, several subgroups showed significant results (P < 0.05) in the univariate meta-regression analysis. Therefore, further research with methodological standardization is necessary to improve accuracy, eliminate heterogeneity, and provide more robust evidence for using CT-based radiomics in predicting LNM in patients with EC.

LASSO regression is widely used for feature selection and dimensionality reduction, aiming to reduce the number of features and eliminate irrelevant ones (36, 37). In the subgroup analysis of this study, using LASSO alone for dimensionality reduction resulted in slightly higher specificity than combining it with other methods. This observation may be attributed to the distribution of data features and the division of subgroups. Most studies on radiomics diagnostic models used LR due to the binary nature of LNM status. Nevertheless, studies utilizing other algorithms, including SVM and RF, showed higher sensitivity rates than those using LR. Additionally, previous studies have shown that neural network models or RF based on clinical features could more effectively predict LNM than traditional LR, exhibiting higher AUC, specificity, positive predictive value, and accuracy (38, 39). Regrettably, owing to limited available literature, only one article each for SVM and RF was retrieved, making it difficult to draw reliable conclusions regarding the comparison between SVM or RF and LR.

Image segmentation is a pivotal element of radiomic analysis, incorporating manual delineation using 2D or 3D images, as well as semi-automatic and fully automatic techniques. However, a universally accepted standard for tumor segmentation remains elusive (40). Although manual segmentation offers high precision, it is labor-intensive, subjective, and lacks standardization, leading to limited reproducibility and elevated time and labor expenses. Semi-automatic segmentation necessitates manual refinement, whereas automatic segmentation employs sophisticated computer algorithms for efficient and reproducible lesion boundary identification (41, 42). However, it’s crucial to mention that the studies incorporated in this research exclusively used manual delineation for image segmentation. Moreover, only one study in this meta-analysis employed the 2D method, and the subgroup analysis did not reveal a significant difference in sensitivity and specificity between the 2D and 3D methods. Nonetheless, the majority of previous studies have recognized that radiomics-based 3D imaging traits offer a wider and more diverse range of specific information, covering the entire tumor volume and providing a more comprehensive and accurate representation of its shape, size, and texture. Furthermore, 3D segmentation enhances reproducibility by reducing interobserver variability and offers a standardized approach to tumor delineation (43, 44). Hence, future research could concentrate on investigating the advantages and limitations of manual, semi-automatic, and fully automatic delineation in both 2D and 3D methods in radiomics-based imaging analysis, with the goal of determining the most suitable imaging technique for specific clinical situations and enhancing the accuracy and reproducibility of radiomics-based tumor characterization.

Previous studies have highlighted the potential impact of variations in manufacturers and devices on the reproducibility of radiomics features, which could affect the precision of image diagnosis (13, 45). Similarly, the subgroup analysis results of this study revealed that radiomics features derived from distinct CT devices had an impact on the pooled specificity. Nonetheless, it is important to interpret these results cautiously, considering the limited number of studies included in the meta-analysis and the potential for bias due to the small sample size. Multicenter studies can validate the generalization ability of radiomics models by overcoming data differences across regions and devices, thereby improving the stability and reliability of the model (46). However, it is worth noting that all the studies included in this analysis were conducted in the same geographical region, China, which introduces a potential geographical bias. To gain a deeper understanding of the value of radiomics in diagnosing LNM in EC, further analysis is required through more prospective, multi-regional, and high-quality studies.

To assess the robustness of our study, we conducted a sensitivity analysis by sequentially removing one literature source at a time. The results showed no significant changes in the combined DOR after each exclusion, indicating that individual studies did not significantly influence our meta-analysis and that the conclusions were stable and reliable. Moreover, the lack of publication bias, as evidenced by Deeks’ funnel plot, further supports the credibility of our findings.

To bolster the robustness and reproducibility of radiomics methodologies, Lambin et al. introduced the RQS guidelines in 2017 (13), aiming to establish a benchmark for quality in radiomics research. However, the absence of standardized quality thresholds remained a notable gap. In response, Wesdorp et al. (47) suggested adopting a 30% cut-off score to enhance clarity and consistency across studies. Despite the RQS percentage of included studies in this meta-analysis ranging from 38.9% to 44.4%, surpassing the 30% threshold, and the pooled diagnostic efficacy demonstrating commendable performance in detecting LNM, the methodological quality of included studies remained a concern. This was because none of the studies utilized phantoms to assess robustness against inter-scansner discrepancies and vendor-specific characteristics. Additionally, comprehensive cost-effectiveness analysis, discussions on potential biological correlations, and a prospective study design were lacking in these studies. Therefore, caution is advised when interpreting the study outcomes.

Several constraints should be considered in the meta-analysis. Firstly, a constrained number of studies met our selection criteria. Secondly, the exclusively retrospective studies analyzed, all conducted in China and solely encompassing English-language publications, may have introduced selection biases and affected quality assessment, thereby potentially constraining the generalizability of our findings. Thirdly, despite conducting various analyses, heterogeneity persisted, emphasizing the need for cautious interpretation of the pooled quantitative results. During data extraction, the highest diagnostic performance model was chosen among multiple models, potentially leading to overestimating the radiomics diagnostic accuracy. Lastly, radiomics could be influenced by factors such as imaging equipment technology and protocols, contributing to heterogeneity. Therefore, establishing standardized presentation protocols in future radiomics research papers is necessary.

## Conclusions

Our findings indicated that the CT-based radiomics demonstrated good diagnostic accuracy in predicting LNM in EC, with commendable sensitivity and specificity levels. However, considering the suboptimal RQS and observed heterogeneity among the included studies, it is essential to conduct additional high-quality, multicenter, and large-scale prospective trials to establish more robust and conclusive evidence for the findings presented in this research.

## Data availability statement

The original contributions presented in the study are included in the article/**Supplementary Materials**, further inquiries can be directed to the corresponding author/s.

## Author contributions

LL: Conceptualization, Data curation, Funding acquisition, Investigation, Methodology, Writing – original draft, Writing – review & editing, Project administration. HL: Data curation, Formal analysis, Investigation, Methodology, Validation, Writing – original draft, Writing – review & editing. YZ: Data curation, Methodology, Software, Writing – original draft, Writing – review & editing. JY: Formal analysis, Writing – original draft, Writing – review & editing. CW: Methodology, Visualization, Writing – original draft, Writing – review & editing. LD: Methodology, Writing – original draft, Writing – review & editing. PX: Software, Writing – original draft, Writing – review & editing. WW: Supervision, Visualization, Writing – review & editing. MX: Supervision, Visualization, Writing – review & editing. DS: Conceptualization, Funding acquisition, Project administration, Supervision, Visualization, Writing – review & editing.
