# Supplementary material for: CT-based radiomics for predicting lymph node metastasis in esophageal cancer: a systematic review and meta-analysis
Source: Front Oncol. 2024 Mar 19;14:1267596. doi: 10.3389/fonc.2024.1267596 (PMC10993774; doi:10.3389/fonc.2024.1267596)
Supplement: Supplementary file 1 [file DataSheet_1.docx]

Supplementary Material

1. **Table S1 The key components of the Radiomics Quality Score (RQS).**
2. **Table S2 The results of individual Radiomics Quality Score (RQS) ratings of each study.**
3. **Table S3 Summary of the QUADAS-2 Assessment of each study.**
4. **Table S4 Results of sensitivity analyses for each study.**
5. **Figure S1 Forest plot of univariable meta-regression and subgroup analyses.**

**Table S1** The key components of the Radiomics Quality Score(RQS).

| **RQS scoring item** | **Interpretation** |
| --- | --- |
| Image protocol quality | +1 for well documented protocols, +1 for publicly available protocols |
| Multiple segmentation | +1 if segmented multiple times (different physicians, algorithms, or perturbation of regions of interest) |
| Phantom study | +1 if texture phantoms were used for feature robustness assessment |
| Imaging at multiple time points | +1 multiple time points for feature robustness assessment |
| Feature reduction or adjustment for multiple testing | −3 if nothing, +3 if either feature reduction or correction for multiple testing |
| Non radiomics | +1 if multivariable analysis with non-radiomics features |
| Biological correlates | +1 if present |
| Cutoff analyses | +1 if cutoff either pre-defined or at median or continuous risk variable reported |
| Discrimination statistics | +1 for discrimination statistic and statistical significance, +1 if resampling applied |
| Calibration statistics | +1 for calibration statistic and statistical significance, +1 if resampling applied |
| Prospective study | +7 for prospective validation within a registered study |
| Validation | −5 if no validation/+2 for internal validation/+3 for external validation/+4 two external validation datasets or validation of previously published signature/+5 validation on ≥3 datasets from >1 institute |
| Comparison to “gold standard” | +2 for comparison to gold standard |
| Potential clinical utility | +2 for reporting potential clinical utility |
| Cost-effectiveness analysis | +1 for cost-effectiveness analysis |
| Open science and data | +1 for open-source scans, +1 for open-source segmentations, +1 for open-source code, +1 open-source representative segmentations and features |

| **Table S2** The results of individual Radiomics Quality Score(RQS) ratings of each study. | | | | | | |
| --- | --- | --- | --- | --- | --- | --- |
| Study Criteria | Chen 2022 | Li 2021 | Ou 2021 | Peng 2022 | Shen 2018 | Yu 2021 |
| Image protocol quality | 1/1/- | 1/1/- | 1/1/- | 1/1/- | 1/1/- | 1/0/1 |
| Multiple segmentation | 1/1/- | 1/1/- | 1/1/- | 1/1/- | 1/1/- | 1/1/- |
| Phantom study | 0/0/- | 0/0/- | 0/0/- | 0/0/- | 0/0/- | 0/0/- |
| Imaging at multiple time points | 0/0/- | 0/0/- | 0/0/- | 0/0/- | 0/0/- | 0/0/- |
| Feature reduction or adjustment for multiple testing | 3/3/- | 3/3/- | 3/3/- | 3/3/- | 3/3/- | 3/3/- |
| Non radiomics | 1/1/- | 1/1/- | 0/1/1 | 1/1/- | 1/1/- | 0/0/- |
| Biological correlates | 0/0/- | 0/0/- | 0/0/- | 0/0/- | 0/0/- | 0/0/- |
| Cutoff analyses | 0/0/- | 0/0/- | 0/0/- | 0/1/0 | 0/0/- | 0/0/- |
| Discrimination statistics | 2/2/- | 2/2/- | 2/2/- | 2/2/- | 2/2/- | 2/2/- |
| Calibration statistics | 0/0/- | 0/0/- | 0/0/- | 2/2/- | 2/2/- | 0/0/- |
| Prospective study | 0/0/- | 0/0/- | 0/0/- | 0/0/- | 0/0/- | 0/0/- |
| Validation | 2/2/- | 2/2/- | 2/2/- | 2/2/- | 2/2/- | 2/2/- |
| Comparison to “gold standard” | 2/2/- | 2/2/- | 2/0/2 | 2/2/- | 2/2/- | 2/2/- |
| Potential clinical utility | 2/2/- | 2/2/- | 2/2/- | 2/2/- | 2/2/- | 2/2/- |
| Cost-effectiveness analysis | 0/0/- | 0/0/- | 0/0/- | 0/0/- | 0/0/- | 0/0/- |
| Open science and data | 1/1/- | 1/0/0 | 1/1/- | 1/0/0 | 1/0/0 | 1/1/- |
| Total scores (Maximum:36) | 15/15/15 | 15/14/14 | 14/13/15 | 17/17/16 | 17/16/16 | 14/13/14 |
| Final RQS percentage | 41.7% | 38.9% | 41.7% | 44.4% | 44.4% | 38.9% |
| Ratings are presented as LSL/YZ/HL. | | | | | | |

| **Table S3** Summary of the QUADAS-2 Assessment of each study. | | | | | | | |
| --- | --- | --- | --- | --- | --- | --- | --- |
| Study | Risk of Bias | | | | Applicability Concern | | |
|  | Patient Selection | Index Test | Reference Standard | Flow and Timing | Patient Selection | Index Test | Reference Standard |
| Chen 2022 | ☺/J/- | ?/?/- | ☺/J/- | ☺/J/- | ☺/J/- | ☺/J/- | ☺/J/- |
| Li 2021 | ?/J/? | ☹/L/- | ☺/J/- | ?/?/- | ☺/J/- | ☺/J/- | ☺/J/- |
| Ou 2021 | ?/J/? | ☺/J/- | ☺/J/- | ☺/J/- | ☺/J/- | ☺/?/? | ☺/J/- |
| Peng 2022 | ☺/J/- | ?/?/- | ☺/J/- | ☺/J/- | ☺/J/- | ☺/J/- | ☺/J/- |
| Shen 2018 | ?/?/- | ?/?/- | ☺/J/- | ☺/J/- | ☺/J/- | ☺/J/- | ☺/J/- |
| Yu 2021 | ?/?/- | ☺/J/- | ☺/J/- | ☺/J/- | ☺/J/- | ☺/J/- | ☺/J/- |
| Assessments are presented as LSL/YZ/HL. L, high; J, low; ?, unclear. | | | | | | | |

**Table S4** Results of sensitivity analyses for each study.

| **Eliminate study** | **Sensitivity (95% CI)** | **Specificity (95% CI)** | **PLR (95% CI)** | **NLR (95% CI)** | **DOR (95% CI)** | **AUC** |
| --- | --- | --- | --- | --- | --- | --- |
| Chen 2022 | 0.73(0.65-0.79) | 0.77(0.67-0.84) | 3.1(2.2-4.5) | 0.36(0.28-0.46) | 9(5-15) | 0.77(0.73-0.81) |
| Li 2021 | 0.74(0.67-0.80) | 0.76(0.67-0.83) | 3.1(2.2-4.3) | 0.34(0.27-0.44) | 9(5-15) | 0.78(0.74-0.82) |
| Ou 2021 | 0.74(0.66-0.81) | 0.78(0.68-0.85) | 3.3(2.3-4.7) | 0.33(0.25-0.44) | 10(6-17) | 0.80(0.77-0.84) |
| Peng 2022 | 0.74(0.67-0.80) | 0.74(0.67-0.79) | 2.8(2.2-3.6) | 0.35(0.27-0.45) | 8(5-13) | 0.80(0.77-0.84) |
| Shen 2018 | 0.73(0.66-0.78) | 0.79(0.73-0.83) | 3.4(2.6-4.4) | 0.35(0.28-0.44) | 10(6-15) | 0.82(0.79-0.85) |
| Yu 2021 | 0.72(0.65-0.79) | 0.76(0.66-0.84) | 3.0(2.1-4.4) | 0.36(0.28-0.46) | 8(5-14) | 0.77(0.73-0.80) |
| PLR, positive likelihood ratio; NLR, negative likelihood ratio; DOR, diagnostic odds ratio; AUC, area under the curve. | | | | | | |


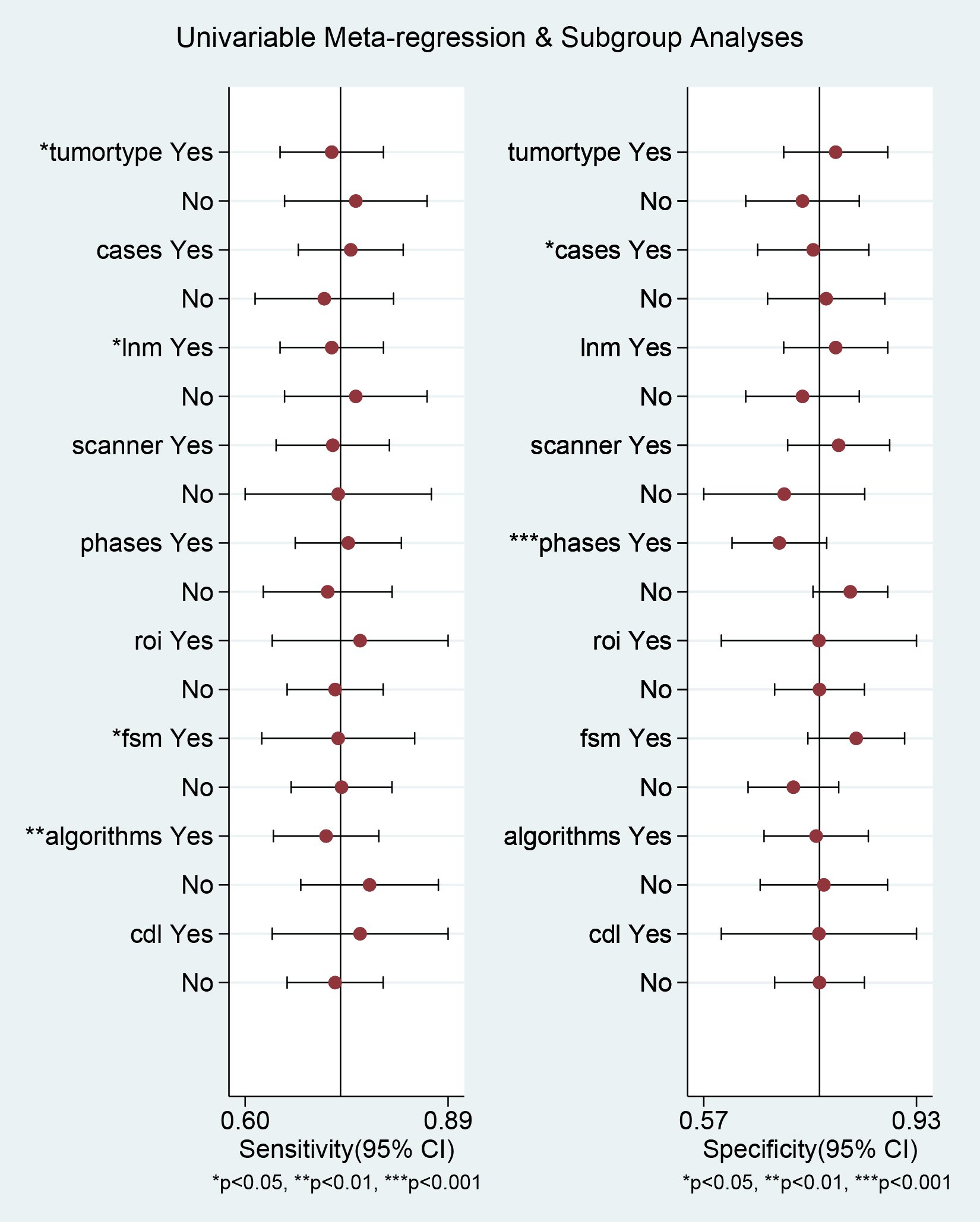


**Figure S1** Forest plot of univariable meta-regression and subgroup analyses. lnm, lymph node metastasis; roi, region of interest; fsm, feature selection method; cdl, combine deep learning.
